# Efficient Dynamic Replication Algorithm Using Agent for Data Grid

**DOI:** 10.1155/2014/767016

**Published:** 2014-03-24

**Authors:** Priyanka Vashisht, Rajesh Kumar, Anju Sharma

**Affiliations:** ^1^Department of Computer Science and Engineering, Amity School of Engineering and Technology, Bijwasan, New Delhi 110061, India; ^2^School of Mathematics and Computer Applications, Thapar University, Patiala 147004, India

## Abstract

In data grids scientific and business applications produce huge volume of data which needs to be transferred among the distributed and heterogeneous nodes of data grids. Data replication provides a solution for managing data files efficiently in large grids. The data replication helps in enhancing the data availability which reduces the overall access time of the file. In this paper an algorithm, namely, EDRA using agents for data grid, has been proposed and implemented. EDRA consists of dynamic replication of hierarchical structure taken into account for the selection of best replica. Decision for selecting the best replica is based on scheduling parameters. The scheduling parameters are bandwidth, load gauge, and computing capacity of the node. The scheduling in data grid helps in reducing the data access time. The distribution of the load on the nodes of data grid is done evenly by considering scheduling parameters. EDRA is implemented using data grid simulator, namely, OptorSim. European Data Grid CMS test bed topology is used in this experiment. The simulation results are obtained by comparing BHR, LRU, No Replication, and EDRA. The result shows the efficiency of EDRA algorithm in terms of mean job execution time, network usage, and storage usage of node.

## 1. Introduction

In recent years, various scientific applications such as biometric, weather forecasting, Large Hardon Collider Computing and so forth produce a large amount of data [1]. These data are accessed by communities and scientists whose computing and storage devices are geographically distributed. The grid technology seems to be a reasonable way to handle the data produced by these experiments and simulations [2]. Grid provides an infrastructure that involves collaborative use of heterogeneous resources in distributed network for cooperative problem solving [3]. The large amount of distributed data with complex computations makes data management in grid environment more challenging. Data grids involve the complete dynamic life cycle of service deployment and provisioning, management, decomposing, and sharing of distributed data [4]. The huge volume of data and calculations involved create new problems regarding the access, processing and distribution of data in data grids [5]. One of the primary issues of data grid is to optimize the access of data so as to reduce the cost of access performance in geographically distributed environment. Data optimization can be achieved by replicating the data. Replication is the process of creating multiple copies of files on distributed sites of grid. Replicas are developed to enhance the availability of data, load balancing among storage elements, and network performance and provide better data access to datasets in the grid environment where failure is more likely to occur. In case data copy crashes, other copies are made available [6]. Optimization of data replication can be done in two ways: short-term optimization and long-term optimization [7]. Short term optimization can be achieved by static replication. In static replication, location of replica is predefined and cannot be changed. Dynamic replication is a long-term optimization technique which aims at reducing average job access time in data grids [8]. Dynamic replication has the advantage over static replication as it can adapt to the changes in the grid environment. In dynamic replication, the replicas can be deleted or created automatically according to the dynamic nature of the grid. The access time depends on where a job is scheduled for execution of file. Therefore, scheduling is important, which helps in assigning job to the node having replica. If the jobs are not scheduled suitably, then the computational resources will be wasted [9]. This results in uneven distribution of resources in which some nodes are overloaded and other nodes are underloaded. Thus, effective scheduling measures help in reducing overall access time through load balancing across multiple nodes [10]. Scheduling is closely related to load balancing and resource allocation. It is concerned with all techniques allowing an evenly distribution of the workload among the available resources in a system [11]. The main objective of a load balancing is primarily to optimize the average response time of the currently executing applications.

## 2. Related Work

Dynamic replication in data grids has been seeking the attention of researchers these days. To model or simulate a data grid, replication technique plays an important role.

Ranganathan et al. [13] proposed a strategy for creating replicas automatically in decentralized peer-to-peer network. The model ensures replica availability with some probabilistic measure. There is no point of failure as the model does not rely on a central monitoring scheme. The disadvantage is that nodes make decisions based on partial information, which sometimes may lead to unnecessary replication.

**Pseudocode 1:**
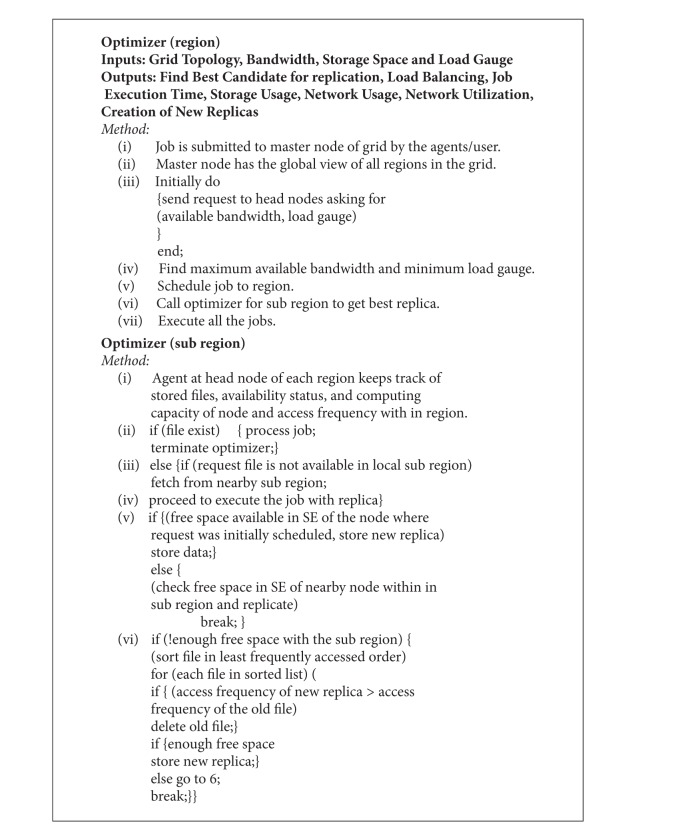
Pseudocode for region optimizer and subregion Optimizer.

Chervenak et al. [14] describe a data grid architectural framework, Giggle (GIGA-scale Global Location Engine), within which a wide range of Replica Location Services (RLSs) can be defined. An RLS consists of the Local Replica Catalogue (LRC) and the Replica Location Index (RLI). The LRC maps logical identifiers to physical locations and vice versa but shares information periodically using soft-state propagation method. Collectively, the LRCs provide a complete and locally consistent record of global replicas. The RLI contains a set of pointers from logical identifiers to LRC. The RLS uses the RLIs to find LRCs that contain requested replicas.

Allcock et al.  [15] developed a replica management service using the Globus Toolkit. The replica management infrastructure includes a replica catalogue and replica management services for managing multiple copies of shared data. The replica catalogue allows users to register files with a logical filename(s) and also maintains mapping between the logical filename and one or more physical locations. It allows users and applications to query the catalogue to find all existing replicas of a file. The replica catalogue was implemented as a Lightweight Directory Access Protocol (LDAP) directory. The management service does not implement the full replica management functionality and does not enforce any replication semantics.

Ranganathan and Foster [16] discuss various replication strategies. They test six different replication strategies: No Replication, best client, cascading, plain caching, caching plus cascading, and fast spread. Various researchers assume user requests as the only parameter to be considered for replica placement and they ignore network latencies. However, network bandwidth plays a vital role in file transfer.

Tang et al. [17] suggested two replication algorithms: Simple Bottom Up (SBU) and Aggregate Bottom Up (ABU) for multitier data grids. The goal of these strategies is to place the replica close to the client. Both strategies decrease the mean response time of data access in comparison with static replication algorithm in multitiered grid. The authors of [18] introduced dynamic replication placement that categorizes the data based on their property. Data is organized into several data categories to which it belongs. This category is used for job scheduling and replication. Then, a job is allocated to a site which has the file in the required category, and this information is used to improve the data replication strategy, which leads to a reduction in the cost of a file transfer. The decision on which file is to be replicated is based on a metric called average number of access.

Lin et al. [19] address the issue of placing database replicas in data grid systems with locality assurance. Each request specifies a workload it requires and a distance within which a replica must be found. Their algorithm selects strategic locations for placing the replica so that the workload among these replicas is balanced, and the service locality required by each data request is guaranteed. They propose two algorithms, MinMaxLoad and FindR. The MinMaxLoad algorithm is used to place replicas in the proper server locations so that the workload on each server is balanced. The FindR algorithm is used to choose the optimal number of replicas and provides service locality. In these algorithms, only tree networks are considered.

Al-Mistarihi and Young [20] discuss replica placement policy and replica selection, which all are embedded in their proposed system in order to reduce job turnaround time, reduce storage cost, and reduce network bandwidth consumption. Their system is termed “Replica Management in Grid” (RmGrid). In RmGrid, there are local optimizers and global optimizers. The local optimizer selects the best replica for the job. The global optimizer gets feedback from the local optimizer such as the replica requests demand and gets some related information from the information provider such as network status. The global optimizer also triggers the replica placement function in order to optimize the number of the replicas and their locations in the grid nodes. The replica placement decision is determined based on location cost which depends on three parameters, namely, (1) sites' power, (2) transfer time, and (3) the replica distribution among the sites.

Afzal et al. [21] propose an approach for scheduling grid which abstracts the details of individual applications, focusing instead on the global cost optimisation problem while taking into account the entire workload, dynamically adjusting to the varying service demands. The algorithm views the grid as a queueing system, seamlessly routing the workflows through the network. The algorithms efficiently schedule applications without requiring performance prediction or negotiation for advance reservations for every stage of the workflow, which leads to significant performance gains. The algorithm also guarantees QoS within required confidence bounds for the end-to-end execution of workflows.

GHS is a long-term, application-level performance prediction and task scheduling system for nondedicated distributed computing. GHS is twofold, in both performance prediction and task scheduling. GHS optimal task scheduling provides a significantly better performance than that of existing methods, while its heuristic scheduling provides a near optimal performance [22].

Rahman et al. [23] used the *P*-median model for the replica placement problem. *P*-median model finds the locations of *P* candidate sites to place a replica that optimizes the aggregated response time. A grid environment is highly dynamic so user requests and network latency vary constantly. Therefore, the candidate sites currently holding replicas may not be the best sites to fetch replica on subsequent requests. The dynamic replica maintenance algorithm is used to reallocate new candidate sites, if a performance metric degrades significantly over last *k* time periods. Here, the value of *P* is constant and this approach will not be suitable for dynamic values of *P*.

A different cost model was proposed by Lamehamedi et al. [24] to decide the dynamic replication. This model evaluates the data access, cost of creation and maintenance of the replicas, and it is applied by the replica manager on each intermediate storage site in a decentralized manner. The data grid structure is a hybrid of tree and ring topologies, and data access among the same tier nodes is allowed. From the simulation results, it is found that the dynamic replication method does not improve the data access performance when relative capacity of the replica server is small, but the detailed configuration parameters are not given.

Park et al. [8] proposed an algorithm called Bandwidth Hierarchy Replication (BHR), which reduces data access time by avoiding network congestions in a data grid network. With the BHR strategy, one can take advantage of “network level locality,” which means that the required file is located in a site that has a large amount of bandwidth between it and the job execution site. In a data grid, some sites may be located within a region where sites are linked closely. If the required file is located in the same region, less time will be consumed for fetching the file. The BHR strategy reduces data access time by maximizing network level locality. Horri et al. [25] proposed the BHR algorithm by using three-level hierarchical structures. They addressed the problem of both scheduling and replication. Sashi and Thanamani [7] proposed a modified BHR algorithm to overcome the limitations of the standard BHR algorithm. The modified BHR region based replication algorithm increases the data availability by replicating files within the region to the region head and also storing them in the site where the file has been accessed frequently. By using this algorithm, mean job execution time can be minimized; the network is used more effectively. There are certain issues with BHR that are considered in EDRA.

- The master node or head nodes rely only on one replica within a region.
- Dynamic nature of the grid is ignored and the assumption is that all the replicas are available throughout.
- Replicas are not consistent.
- There is no scheduling strategy considered for load balancing.

## 3. Various Issues in Data Replication That Are Addressed in EDRA

Dynamic replication is an optimization technique which aims to increase network bandwidth and availability of data and reduce total access time by considering different issues. The abovementioned issues have been addressed in the proposed algorithm, that is, EDRA (Efficient Dynamic Replication Algorithm) using agents that needs to be addressed before replicating.

*Replica Creation.* Data replication involves decisions like when to create a replica and how many copies of a replica are required. In EDRA, replica is created if the file is not available on the node where the request has been scheduled.

*Replica Placement.* After creating a replica, the main objective is to decide where to place the replica so as to get the fast access and less access latency. The replica in EDRA is placed on the basis of the popularity of the file and the available storage space on the node. The popularity is based on the access frequency of the file on the node.

*Replica Selection.* After placement, the next step is to select the best replica among the pool of available replicas. The criterion for choosing the best replica in EDRA is based on workload of the node, availability status, available bandwidth, and computing capacity of the node.

*Storage Space.* Before the placement of replica, the amount of storage space should be taken into account. If less storage space is available, then there should be some replacement strategies involved like LRU. In EDRA, the storage space is calculated in (1) and the file having less access frequency is replaced by the replica of the file which is being requested.

*Adaptability.* The data replication strategy must be adaptive to the dynamic nature of the grid in order to provide better results. In EDRA, if file is not available at the time of execution of job, then replica of file is created so that grid can adapt according to its dynamic nature.

## 4. Terminologies Used in EDRA

Different entities considered in EDRA are described below.

*Grid Node.* It is the smallest unit of data grid which consists of three components, that is, computing element, storage elements, and replica management as shown in Figure 2.

- *Computing Element *(CE). It provides CPU cycles for the execution of job/request made by user in data grid. Each CE is located on a grid node within the data grid.
- *Storage Element *(SE). It provides storage capacity to store the data produced or used by the users in data grid environment. The amount of storage space available for grid jobs varies over time depending on local storage management policies.
- *Replica Management *(RM). Replica management maintains the replica catalogue where the file information is stored [26]. It provides solution for replica scheduling, monitoring, placement, selection, and so forth. The nodes use RM for scheduling the jobs and collecting and managing information of the replicas.

**Figure 1:**
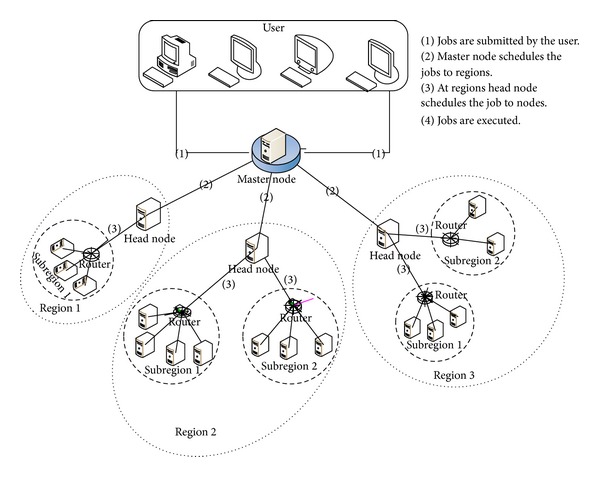
Pictorial representation of grid environment including region, subregion, master node, and head node.

*Master Node.* It facilitates job submission with an interface and helps in scheduling and monitoring the job. Master node contains the global view of all the regions in the data grid. Master node is shown in Figure 1.

*Head Node.* The head node has the local information of the region which helps in executing and scheduling the request at region level as shown in Figure 1. The master node and head nodes in data grid use RM component of data grid node. The rest of the data grid nodes use only storage and computing element.

*Load Gauge.* It is the length of the queue at master node and head node where jobs are waiting to get executed.

*Agent.* An agent is a software or user which works intelligently in the network, according to the information gathered and stored on the node.

*Availability.* This parameter is to check whether a node is available at the time of execution of request or not.

*Access Frequency.* It is the measure of number of file requests made by users running on the nodes in the region [8]. Access frequency helps in deciding the popularity of the file based on the access history. The higher the access frequency of a file on a node is, the more popular the file is.

*Computing Capacity.* This helps in scheduling the jobs to the node having more computing power. The value of available computing capacity is calculated using (2).

*Subregion.* A number of grid nodes collectively form one subregion as shown in Figure 1.

*Region.* A collection of subregions creates a region as shown in Figure 1.

**Figure 2:**
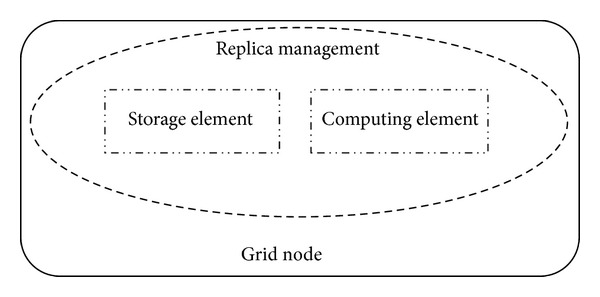
Pictorial representation of grid node.

To illustrate the role of entities in grid environment, an assumption has been made by considering the hierarchical structure of Internet scenario used these days. It is made of many wide and local area networks joined by connecting devices and switching stations. The services of Internet Service Providers (ISPs) are used by end users at different levels. There are international service providers, national service providers, regional service providers, and local service providers. At the top of the hierarchy are the international ISPs to connect nations together [27]. Here, international ISPs are considered as master node. The national ISPs are maintained by some companies. In EDRA algorithm, the national ISPs are similar to regions, and companies can be considered as the head nodes which are responsible for the maintenance of the region. Regional ISPs are small ISPs connected to one or more national ISPs. At the third level, the subregions are considered as regional ISP which is connected to head node of region, that is, national ISP. Local ISPs provide direct service to end users. The local ISPs can be connected to regional ISP or national ISP. Local ISP is entity that just provides internet services. This is like the node in data grid where the file or the replica of the file is placed which is being requested by the user. Pictorial representations of basic entities are shown in Figures 1 and 2.

## 5. Description of EDRA Algorithm

In EDRA, the following assumptions have been made.

- Bandwidth of nodes is the same as within a subregion.
- Bandwidth between two subregions is slightly less than the bandwidth between the nodes within the subregion.
- Bandwidth between two different regions is lowest amongst all the bandwidths allotted in the whole grid.
- There can be only one replica corresponding to the file that can exist in the subregions and is placed on the grid node based on the popularity of the file.

The first three assumptions cause the hierarchy of bandwidth in the data grid. The intra-subregion has broader bandwidth than inter-subregion which effectively minimizes the access time.

The fourth assumption implies that the number of replicas is directly proportional to the number of subregions; that is, number of replicas cannot be more than the number of subregions within a region.

The EDRA algorithm is divided into two parts, that is, region optimizer and subregion optimizer. Region optimizer is executed at the master node of the data grid where the agent helps in scheduling the job to the region based on the load gauge of head node and network bandwidth between master node and head node. This helps in getting the global view of all the regions in the data grid. The subregion optimizer is invoked by master node based on the information gathered by agent at master node to get the best replica. The subregion optimizer at head node of the region has the local view of all the subregions in that region. The local information includes availability status, access frequency, computing capacity, and stored files at a node in the subregion. These parameters help in scheduling the job to the final destination (file/replica) stored at the node of the region by efficient usage of the resources. The pseudocode for region optimizer and subregion optimizer is shown in Pseudocode 1.

## 6. Working of EDRA Algorithm

In EDRA, the request is submitted by the user to the master node, where an agent is placed having the global view of the whole data grid. The jobs are submitted randomly. The agent located at master node schedules the job submitted by user to the regions, where the replica/file has been stored with the goal of improving the overall throughput of grid. The available bandwidth between the master and head nodes of region and the load gauge of the head node is checked. The load gauge is a pointer to the waiting queue of head nodes which tells the number of jobs waiting for their turn to get executed. Available bandwidth and network gauge decide on which region the job is to be scheduled. This also helps master node to balance the load on the regions.

The head node located in regions is responsible for scheduling the jobs to the nodes where the replica is placed. An agent is placed on each head node of the region, which keeps information of the nodes located in the subregions. The information stored on the head nodes is local to the region like availability of the node, access frequency, computing capacity of node, and so forth, within region.

The replica is placed on the nodes based on two factors: high data access frequency and sufficient storage capacity. The high access frequency shows the popularity of the file based on the access history stored at head node. The popularity of the file can be decided by the number of requests made for the file while the jobs are getting executed on the nodes within the region. The available storage capacity in EDRA is based on the storage usage of the node.

The available storage capacity Aval_str_ on a node is calculated as

(1) $$\begin{array}{c} {\text{Aval}}_{\text{str}}=S_{\text{reg}\;}-S_{\text{usage}}, \end{array}$$

where *S*_reg_ is maximum storage capacity of a node and *S*_usage_ is storage space consumed by the node.

If the available storage space has more capacity than the size of the file, the replica is placed on the node. If the node has insufficient space, then old files or replicas are deleted from the node. For deleting a file from the node, the access frequency of new replica is compared with the access frequency of old file stored on the node. The file having less access frequency is deleted and is replaced by the new replica. The respective information of the replicas is updated on the replica catalogue.

At the time of selection of the replica, the agent checks the current available status of the node, computing capacity of the node for execution of request, and maximum available bandwidth and load gauge. The computing capacity of the node is the factor which decides how fast a job can be processed by the node. The available computing capacity Aval_cap_ of the node is calculated as

(2) $$\begin{array}{c} {\text{Aval}}_{\text{cap}}={\text{Comp}}_{\text{node}}-{\text{Comp}}_{\text{usage}}, \end{array}$$

where Comp_node_ is node's CPU computing capacity (MHz) and Comp_usage_ is CPU usage of node.

If file is available in the subregion, the job is processed; otherwise, agent sends the job to the neighbouring subregion. After scheduling the request to the neighbouring subregion, the job is processed on the neighbouring node. The information of replica in replica catalogue at head node is updated by the agent of head node.

After processing the job on neighbouring node, the replica is created in the subregion where initially the job was scheduled. The replica catalogue is updated and the job is executed.

Though the number of replicas of the file has been increased within a region by the EDRA algorithm, this consumes more storage space as compared to BHR. By increasing the number of replicas of the file, the availability of the file has been increased, which helps in reducing the data access time of the file. There is always a tradeoff between the storage and the access time.

## 7. Testbed and EDRA Implementing Using OptorSim

**Figure 3:**
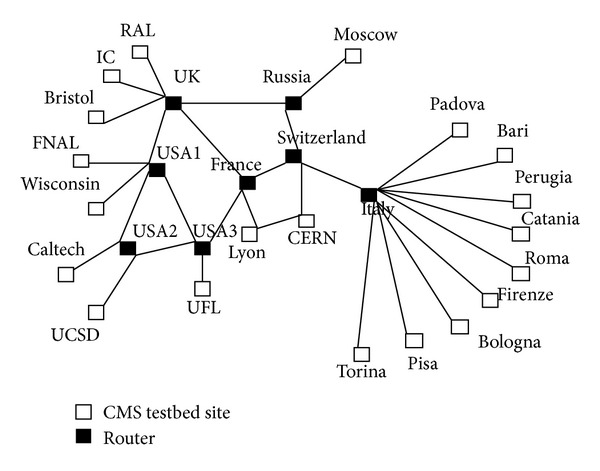
CMS topology in simulation [12].

For EDRA, European Data Grid CMS testbed architecture has been considered [12]. CERN and FNAL act like master nodes as initially data is produced on these nodes. Two master nodes are considered so as to share the workload. Moreover, in case of failure of one master node, another will take the responsibility. Jobs are processed on nodes having computing and storage elements. The routers in the architecture used to route the request forward to other nodes in the grid. The storage capacity of master node is 200 GB, and the storage capacity of all the other nodes is 50 GB. The size of file is 1 GB each. Existing replication strategies commonly assume that data is read only. There are maximum 500 jobs with 10 job types. The jobs considered here were Job1, Job2,…, and so on till Job10 in job table of the job configuration file and we referred to these jobs as different job types. Here, the jobs are chosen “at random” but weighted by their selection probability. The topology of CMS testbed is shown in Figure 3. Padova, Bari, and Perugia collectively constitute one subregion. Similarly, Catania, Roma, Firenze, and Bologna constitute another subregion and the remaining are subregion 3 for Italy, similarly for USA, UK, and so forth.

The agent is not depicted in Figure 3 since it is not a physical entity and is located at the master node and head node, which helps in scheduling the jobs to the regions and subregions, respectively.

EDRA is written in Java and is integrated in Optor package of simulator on Windows platform. This algorithm is then included in a parameter file so that it can be selected while testing using OptorSim's Graphical User Interface (GUI). The parameters considered are access time, effectiveness of network usage, and the storage capacity.

The simulation and implementation of EDRA are done on OptorSim. OptorSim [26] provides a framework suitable for the simulation of realistic grid scenarios and the evaluation of grid optimisation algorithms. OptorSim was developed by the European Data Grid projects [12] to test the dynamic replication strategies.

The input can be controlled by using four configuration files, namely, the grid configuration file, parameter configuration file, job configuration file, and bandwidth configuration file.

The grid configuration file specifies the network topology. The parameter configuration file sets the parameters like number of jobs, access pattern of the job, choice of replication, and so forth. The number of jobs considered here is 100, 200, 300, 400, and 500. The access pattern taken is Random Zipf. The job configuration file has information for the simulated jobs, whereas the bandwidth configuration file gives the background network traffic.

In OptorSim, various job execution scenarios already exist. For EDRA, different job execution scenarios have been taken into consideration as shown in Table 1.

Different performance metrics that are used for EDRA are shown in Table 2.

## 8. Results

The efficiency of EDRA is calculated based on three Performance Metrics, that is,* mean job execution time*,* effective network usage*, and* storage usage *of the nodes. EDRA is compared with three other dynamic replication strategies, that is, BHR, No Replication, and LRU (Least Recently Used) on the OptorSim simulator using the abovementioned Performance Metrics. At the time of simulation in No Replication strategy, the complete data is available at the root of the hierarchy. In case of LRU, file is replicated when it is needed [13]. In BHR, there exists only one replica in a region based on the popularity of the file. In EDRA, the number of replicas depends on the number of subregions. In the next section, the detailed comparison of LRU, BHR, No Replication, and EDRA has been done.

### 8.1. Mean Job Execution Time (MJET)

The mean job execution time is calculated as time to execute a file, the time spent by a job in waiting queue divided by the number of jobs completed. It can be represented as

(3) $$\begin{array}{c} \text{MJET}=\frac{\sum_{i=1}^{n}\left(T_{i}+W_{i}\right)}{n}, \end{array}$$

where *n* is the number of jobs processed by the system, *T*_*i*_ is the time to execute the *i*th job, and *W*_*i*_ is the waiting time of *i*th job that has been spent in the queue.

**Figure 4:**
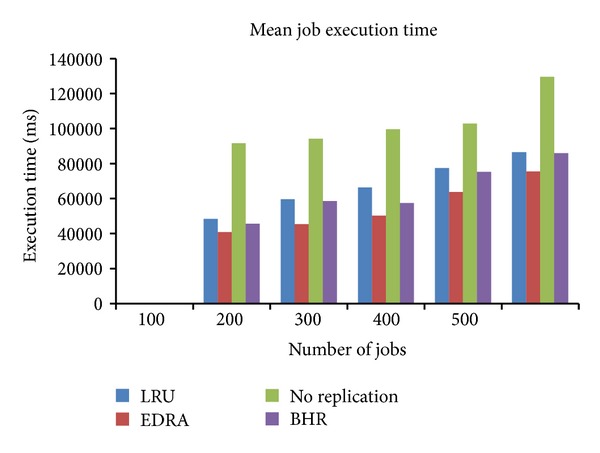
Comparison of mean job execution time with existing algorithms.

The EDRA along with LRU, No Replication, and BHR was tested using different job numbers of 100, 200, 300, 400, and 500 jobs. The job execution time for Random Zipf Access Pattern Generator is shown in Figure 4.

It is evident that as the number of jobs increases, EDRA is able to process the job in the lowest mean execution time as shown in Figure 4. EDRA is able to access the file in less time, which reduces the waiting time and increases the availability. Moreover, the scheduling strategy used is able to process the workload in less time.

The No Replication strategy performs worse in all the cases. BHR performs better than LRU and No Replication.

### 8.2. Effective Network Usage (ENU)

The replication process of a file needs network bandwidth to access a file. The network usage to access a file is calculated as

(4) $$\begin{array}{c} \text{ENU}=\frac{\left(N_{\text{remfile}\;}+N_{\text{filerep}}+N_{\text{locfile}}\right)}{\left(N_{\text{time}\_\text{remfile}}+\;N_{\text{time}\_\text{filerepe}}\right)}, \end{array}$$

where *N*_remfile_ is the number of times the computing element reads file from storage element on different regions multiplied by size of file, *N*_filerep_ is the total number of file replications that take place during job execution multiplied by the size of the file, *N*_locfile_ is the number of times the computing element reads file from storage element on the same subregion or region multiplied by size of file, *N*_time_remfile_ is the time taken to access the remote file, and *N*_time_filerepe_ is the time taken to replicate the file.

**Figure 5:**
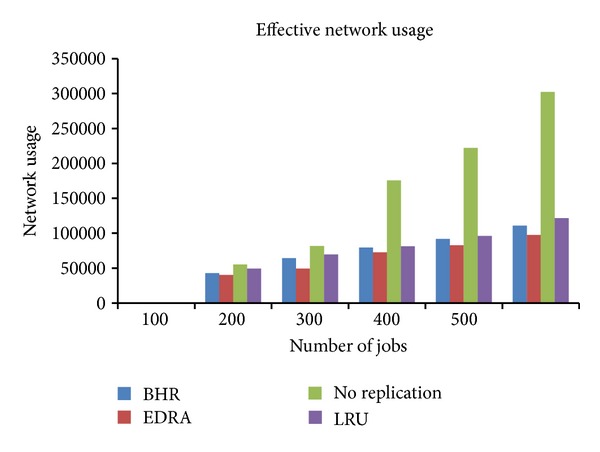
Comparison of effective network usage.

The comparison of four strategies, that is, EDRA, BHR, No Replication, and LRU, is shown in Figure 5.

According to the assumption number (i) mentioned above, the file within a local network has the maximum bandwidth, so time to access the local file is ignored. No Replication strategy consumes maximum network bandwidth as shown in Figure 5. The effective network usage is better in LRU. As compared to BHR, EDRA shows better results; that is, it consumes less network bandwidth. The availability of the file increases at local level by increasing the number of replicas. This reduces file transfer time which results in less network usage while accessing the file.

### 8.3. Storage Used (SU)

The percentage of storage used (SU) by files in MB is specified by storage used within region under this strategy. This can be calculated as

(5) $$\begin{array}{c} \text{SU}=\frac{\left(S_{\text{total}}-\sum_{i=1}^{n}\text{Ava}{\text{l}}_{\text{str}}\right)}{S_{\text{total}}}*100, \end{array}$$

where *S*_total_ is the total storage capacity of a region and Aval_str_  is calculated from (1).

**Figure 6:**
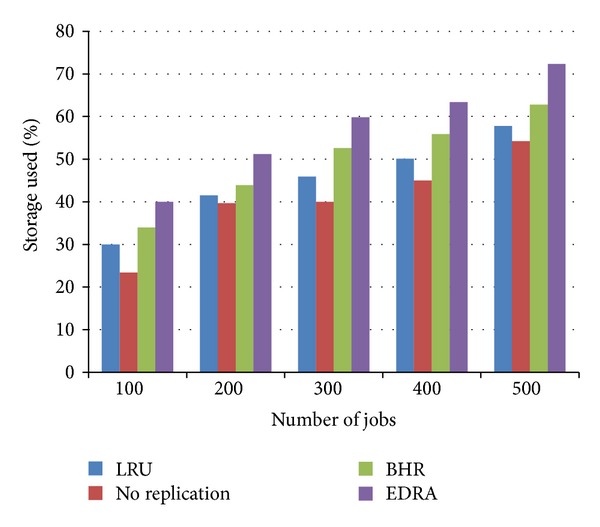
Comparison of storage usage.

The storage used in No Replication strategy is the best as there is no replica created. EDRA performance is the worst as the number of replicas has increased. The LRU and BHR strategies use moderate storage space. The results are depicted in Figure 6.

## 9. Conclusion

This paper presents the EDRA (Efficient Dynamic Replication Algorithm) using agents in data grids for optimization of replication in grid environment. In this paper, the issues of BHR algorithm have been addressed. The main issue in BHR is that the algorithm considers one replica corresponding to each region. The proposed algorithm increases the availability by placing one replica in each subregion. This helps in increasing the number of replicas within a region. The number of replicas is increased at the cost of storage capacity. In the previous works based on BHR, the assumption was that all the nodes are available all the time. Due to dynamic nature of grid, this assumption is not appropriate. The proposed algorithm checks the availability status of a particular node within region and takes the decision accordingly. To increase the performance of EDRA, twofold scheduling policy has been adapted first at master node and second at head node to increase the performance of the algorithm. The scheduling has incorporated load balancing factor which results in better execution of jobs.

## 10. Future Work

Various strategies have been used in data grid to overcome the challenges in dynamic data replication. In this paper, replicas considered in the experiment are read only. In future, the writable replicas can be considered. The writable replica opens a room for consistency of replicas. The consistency will help in improving the overall system performance.

## Figures and Tables

**Table 1:** Job execution scenarios.

Job execution scenarios	Values
Number of jobs	100, 200, 300, 400, 500
Number of job types	10
Size of single file	1 GB
Scheduling algorithm used by Resource Broker	Random
Choice of access pattern	Random Zipf

**Table 2:** Performance Metrics.

Performance Metrics	Description
Mean job execution time (milliseconds)	Time to execute the job + waiting time/number of jobs completed
Effective network usage	Specifies the network utilization
Storage used (MB)	Specifies spaces used by files
